# Proton Pump Inhibitors Inhibit Pancreatic Secretion: Role of Gastric and Non-Gastric H^+^/K^+^-ATPases

**DOI:** 10.1371/journal.pone.0126432

**Published:** 2015-05-18

**Authors:** Jing Wang, Dagne Barbuskaite, Marco Tozzi, Andrea Giannuzzo, Christiane E. Sørensen, Ivana Novak

**Affiliations:** Department of Biology, Section for Molecular Integrative Physiology, August Krogh Building, University of Copenhagen, Copenhagen, Denmark; RWTH Aachen, GERMANY

## Abstract

The mechanism by which pancreas secretes high HCO_3_^-^ has not been fully resolved. This alkaline secretion, formed in pancreatic ducts, can be achieved by transporting HCO_3_^-^ from serosa to mucosa or by moving H^+^ in the opposite direction. The aim of the present study was to determine whether H^+^/K^+^-ATPases are expressed and functional in human pancreatic ducts and whether proton pump inhibitors (PPIs) have effect on those. Here we show that the gastric HKα1 and HKβ subunits (*ATP4A*; *ATP4B*) and non-gastric HKα2 subunits (*ATP12A*) of H^+^/K^+^-ATPases are expressed in human pancreatic cells. Pumps have similar localizations in duct cell monolayers (Capan-1) and human pancreas, and notably the gastric pumps are localized on the luminal membranes. In Capan-1 cells, PPIs inhibited recovery of intracellular pH from acidosis. Furthermore, in rats treated with PPIs, pancreatic secretion was inhibited but concentrations of major ions in secretion follow similar excretory curves in control and PPI treated animals. In addition to HCO_3_^-^, pancreas also secretes K^+^. In conclusion, this study calls for a revision of the basic model for HCO_3_^-^ secretion. We propose that proton transport is driving secretion, and that in addition it may provide a protective pH buffer zone and K^+^ recirculation. Furthermore, it seems relevant to re-evaluate whether PPIs should be used in treatment therapies where pancreatic functions are already compromised.

## Introduction

Digestive processes along the gastrointestinal tract are aided by acidic and basic secretions by a number of epithelia. In particular, the pancreas and the stomach are the most avid base (HCO_3_^-^) and acid (H^+^) secretors, respectively. The gastric H^+^ secretory mechanisms are well established, however, the cellular mechanism by which pancreatic duct cells secrete almost isotonic HCO_3_^−^ fluid has long been a challenge to epithelial physiologists.

The current ion transport model for pancreatic HCO_3_^−^ secretor, the duct cell, involves two machineries on the two epithelial membranes: first, cells accumulate cellular HCO_3_^−^ with the help of a basolateral Na^+^-HCO_3_^−^ cotransporter (pNBC, NBCe1) and a Na^+^/H^+^ exchanger (NHE1) together with carbonic anhydrase; second, HCO_3_^−^ efflux occurs via co-operation between Cl^−^ channels and Cl^−^/HCO_3_^−^ anion exchangers from the SLC26A6 family, e.g. SLC26A6 on the luminal membrane [1]. The Cl^−^ channels are the cystic fibrosis transmembrane conductance regulator (CFTR) Cl^−^ channels, which may have some HCO_3_^−^ permeability [2,3] and the Ca^2+^-activated Cl^−^ channels, such as TMEM16A/ANO1 [4]. Furthermore, K^+^ channels (e.g. K_Ca_3.1, K_Ca_1.1, KCNQ1) maintain the membrane potential and provide the driving force for anion secretion together with the Na^+^/K^+^-ATPase [5–7]. Na^+^ and water follows passively. Nevertheless, this model can only explain production of 80–100 mM NaHCO_3_ in secreted fluid, yet human pancreas can secrete up to 140 mM NaHCO_3_. In addition to NBCs and NHE, earlier studies have shown vacuolar H^+^ ATPase (V-ATPase) activity on the basolateral membrane of pancreatic ducts by intracellular pH (pH_i_) measurements and use of the V-ATPase inhibitor bafilomycin A_1_ [8–11] Nevertheless, whether the V-ATPase plays a significant role in pancreatic HCO_3_^−^ secretion is not clarified, as for example in guinea pig pancreatic ducts bafilomycin A_1_ could not inhibit agonist-stimulated HCO_3_^−^ and fluid secretion [12,13]. Therefore, in the present study we have focused on the function of H^+^/K^+^-ATPases (pumps), which are pharmacologically approachable and physiologically relevant. Such H^+^/K^+^-pumps have not been proposed for HCO_3_^−^ secreting tissues, except for our earlier study on rat pancreatic ducts [14]; rather they have well-established roles in acid secretion, and in H^+^ and K^+^ homeostasis in other tissues.

The H^+^/K^+^-ATPases are classified into two subfamilies, gastric and non-gastric (latter also called colonic), coded by *ATP4A* and *ATP12A*. The gastric H^+^/K^+^-ATPase is expressed in stomach parietal cells, kidney distal nephrons [15–17] and cochlea [18,19], where they are responsible for H^+^ secretion, K^+^ absorption and K^+^ recirculation, respectively. The non-gastric H^+^/K^+^-ATPase is present in several epithelial tissues including colon, kidney, skin, placenta, and prostate, and here it is associated with acid-base or K^+^ and Na^+^ homeostasis [17,20–22]. Each pump is composed of two catalytic α-subunits and two regulatory β-subunits. The gastric α-subunit (HKα1) assembles with the gastric β-subunit (HKβ), while the non-gastric α-subunit (HKα2) can borrow the gastric β-subunit, and β3/β1-subunits of the Na^+^/K^+^-ATPase [20,23–25]. The gastric H^+^/K^+^-ATPase is the primary target for treatment of peptic and duodenal ulcers and reflux diseases [26]. Proton pump inhibitors (PPIs), such as omeprazole, are activated in acid environment of secretory canaliculus of the parietal cells and bind covalently to cysteines of the ATPase [26]. Another experimental class of ATPase inhibitors are potassium-competitive acid blockers (P-CABs), such as SCH28080, though at high concentrations they may also inhibit the non-gastric H^+^/K^+^-ATPase [27,28].

Our hypothesis is that the H^+^/K^+^-ATPases may be important in supporting pancreatic function, which may be of particular relevance in human pancreas. In a most simplistic way, one could envisage that these ATPases would pump H^+^ out towards the interstitium and provide HCO_3_^-^ for luminal transport and thus fluid secretion.

Thus the aim of this study was to establish whether human pancreatic ducts express functional gastric and/or non-gastric H^+^/K^+^-ATPase and whether H^+^/HCO_3_^-^ transport and whole pancreatic secretion is sensitive to proton pump inhibitors (PPIs). For this purpose we have used human cells/tissue and performed *in vivo* studies on the rat pancreas where H^+^/K^+^-ATPases are expressed, as already established in our earlier study [14]. We show that human duct cells express subunits of both gastric and non-gastric H^+^/K^+^-ATPases and these exhibit unusual localization patterns. We propose that these pumps have physiological functions in pancreatic H^+^/HCO_3_^-^ transport and fluid secretion, and speculate on their additional role in mucosal protection and K^+^ recirculation. Most importantly, the present studies show that proton pump inhibitors inhibit pancreatic secretion and we speculate about consequences of using these drugs as treatment therapies in several pancreatic diseases.

## Materials and Methods

### Ethical Approval

The permission for animal experiments and protocols were approved by the Danish Animal Experimentation Inspectorate (license no. 2011/561-56 and 2012-15-2934-00693). The experiments were performed on male Wistar rats weighing between 250 and 400 g. Data from 22 rats treated with omeprazole, 6 rats treated with SCH28080, and 21 matched controls were included in the study. For details see below.

### Cell Culture

Pancreatic cell lines (human pancreatic ductal adenocarcinoma) were purchased from ATTC (Manassas, VA, USA). PANC-1 (#CRL-1469) was grown in Dulbecco’s Modified Eagles Medium (DMEM), CFPAC-1 (#CRL-1918) and Capan-1 (#HTB-79) were grown in Iscove’s Modified DMEM (IMDM). Cell culture media contained Glutamax, 10% (20% for Capan-1) fetal bovine serum (FBS), 100 U/ml penicillin and 100 μg/ml streptomycin. Cells were grown at 37°C in a humidified atmosphere with 5% CO_2_. Cells from passage 12 to 25 were used in this study. All standard chemicals were purchased from Sigma-Aldrich unless otherwise stated.

### pH_i_ Measurements

Methods for pH_i_ measurements in Capan-1 cells are based on the one described elsewhere [29]. Briefly, Capan-1 cells grown on standard Ibidi μ-Dish^35mm^ were loaded with 2 μM BCECF/AM (Invitrogen) for 20–30 min; thereafter they were superfused at 2 ml/min, at 37°C. Control perfusate had the following composition (in mM): Na^+^ 145, Cl^−^ 145, K^+^ 4, Ca^2+^ 1.5, Mg^2+^ 1, phosphate 2, HEPES 10 glucose 5; pH was 7.4. For ammonium pulses 20 mM Na^+^ was replaced with NH_4_^+^. In Na^+^-free solutions, N-methyl-D-glucamine was used for replacement. Intracellular pH (pH_i_) was estimated from changes in the fluorescence emission (at 510 nm) from 15–20 cells after excitation at 495 and 440 nm. Signals for each batch of cells were calibrated *in situ* with 1 μm ionophore carbonyl cyanide m-chlorophenyl hydrazone, CCCP, and the fluorescence ratios and pH_i_ were fitted to a calibration curve. A standard method of ammonium pre-pulse was used to study H^+^ transport. Tissues were exposed to ammonium pulses (2–3 min), then ammonium was removed, and pH_i_ recovery rates from acidosis were determined from the initial slopes of pH_i_ changes and expressed as *dpH/dt* (*i*.*e*. pH units/min). The following common representative acid blockers (PPIs and P-CABs) were used: omeprazole (10 μM) and SCH-28080 (10 μM) (Sigma Aldrich). Acidified ethanol (0.15 M HCl in 75% ethanol) was used to prepare omeprazole stock solutions for these experiments, as omeprazole needs acid environment to be activated.

### Reverse transcription polymerase chain reaction (RT-PCR) and real-time PCR

RT-PCR and real time PCR were carried out as detailed in our recent work [4]. Briefly, cells were cultured to confluence and then RNA was isolated with RNeasy Mini Kit (Qiagen 74104). DNase 1 (RNase free DNase Set, Qiagen 79254) was used to avoid any DNA contamination. 1 μg RNA per reaction was used in OneStep RT-PCR Kit (QIAGEN 210212) with amplification parameters as follows: one cycle at 50°C for 30 min and one cycle at 95°C for 15 min followed by 40 cycles at 95°C for 30 s, 55°C for 30 s, 72°C for 1 min, and finally, one cycle at 72°C for 10 min.

For real-time PCR, cDNA was synthesized based on 5 μg RNA template per reaction using the RevertAid First Strand cDNA synthesis kit (Fermentas #K1622) with oligo (dT)18 primers and RevertAid M-MuLV reverse transcriptase, and then purified using GenElute PCR Clean-Up Kit (Sigma, NA1020). The purified cDNA was quantified by absorbance at 260/280 nm, and 100 ng was used as template for each PCR reaction. The PCR reactions were run using LightCycler 480 SYBR Green I Master (Roche, 04707516001) with parameters as follows: pre-incubation for 5 min at 95°C followed by 45 amplification cycles of 10 s at 95°C, 1 min at 55°C, and 30 s at 72°C. A melting curve was performed following the PCR by 5 s at 95°C, 1 min at 65°C, and subsequent heating up to 97°C, and then cooling down for 10 s at 40°C. Reactions were performed as triplicates and were repeated four times. Table 1 shows primers used; these were synthesized by MWGBiotech or TAG Copenhagen A/S (Copenhagen, Denmark). Four house-keeping genes, 18S ribosomal RNA (18SrRNA), β-actin, β-glucuronidase (GUSB) and glutaminyl-tRNA synthetase (QARS) were used for normalization. These genes have relatively stable expression in both normal and cancerous pancreas [30].

**Table 1:** Primer sets used for human pancreatic duct cell lines in RT-PCR and real-time analysis.

Primers	Sequence	Product length
**HKα1 FW**	AAGATCTGCAGGACAGCTACGG	200
**HKα1 RW**	CTGGAACACGATGGCGATCA	
**HKα2 FW**	CCCTGGGAGCTTTCCTTGTGTA	339
**HKα2 RW**	TTCCGAGGCCATAGGAGAGGAT	
**HKβ FW**	GGCCTTCTACGTGGTGATGAC	136
**HKβRW**	CCCGTAAACATCCGGCCTTA	
**18S rRNA FW**	TCGGAACTGAGGCCATGATT	150
**18S rRNA RW**	CGAACCTCCGACTTTCGTTCT	
**β actin FW**	GTGACATTAAGGAGAAGCTGTGC	300
**β actin RW**	CAATGCCAGGGTACATGGTG	
**GUSB FW**	ACTGACACCTCCAAGTATCCCA	200
**GUSB RW**	AACAGGTTACTGCCCTTGACAG	
**QARS FW**	CCTCTATGAGCGACTATTCCAGC	200
**QARS RW**	GATGGCTGTCTGGATCCACG	

### Western Blot Analysis

Pancreatic cells, stomach and colon from mice were lysed by adding 5x diluted lysis buffer (50 mM Tris-base, 0.25 M NaCl, 10 mM EDTA, 0.5% NP40, 1% TritonX-100, 4 mM NaF, pH 7.5). The final lysates were centrifuged at 15,000 g, 4°C for 15 min. Protein concentration was estimated using Coomassie protein assay (Thermo Scientific). Mouse stomach and colon were used as positive control. All solutions contained 1× Sigma protease inhibitor (Sigma S8820). Protein samples were loaded on 10% polyacrylamide precast gels (Invitrogen), separated by electrophoresis, and blotted to PVDF membranes (Invitrogen). Membranes were blocked with 5% skim milk solution in TBS-Tween (0.1%) buffer for 1 h at room temperature and incubated overnight at 4°C with primary antibodies against gastric HKα1(1:1000 rabbit monoclonal, Abcam, EPR12251 or polyclonal Calbiochem 119101), HKβ (1:1000 mouse monoclonal 2G11, Sigma, A274), non-gastric HKα2 (1:1000 rabbit polyclonal, Sigma, HPA039526) or antibody (C384-M79) [28], kindly donated by J. J. H. H. M. De Pont and H. G. P. Swarts, and β-Actin (1:1000 mouse monoclonal C4, Santa Cruz, Sc-47778). Blots were then incubated with appropriate HRP-conjugated antibodies (DAKO or Santa Cruz), developed with EZ-ECL (Biological Industries) and visualized on Fusion FX (Vilber Lourmat) and band intensity was calculated using Bio1D software.

### Immunocytochemistry

Human duct cells (Capan-1) were cultured to confluence on coverslips or Transwells (Costar, 3407), and then fixed in 4% paraformaldehyde for 15 min at room temperature. Human pancreatic sections (GeneTex) were deparaffinized according to standard procedures, and antigen retrieval was performed with 1x citrate buffer in 98°C for 20 min. After washing with PBS, monolayers or pancreatic sections were treated with 0.1 M TRIS-glycine (pH 7.4) for 15 min; washed in phosphate buffered saline, PBS, with 0.3% TritonX-100 and blocked with 10% BSA for 30 min. Subsequently, preparations were incubated with 1:50 to 1:300 dilutions of the primary antibodies recognizing the gastric HKα1, HKβ, or non-gastric HKα2 overnight at 4°C. Then preparations were incubated with secondary antibodies conjugated to Alexa 568 or Alexa 488 (Invitrogen, 1:400). 4’, 6-diamidino-2-phenylindole (DAPI, 1:400) was used for nuclear staining, and a fluorescent dye Texas Red-X phalloidin (Invitrogen; 1:40) was used for actin staining. Fluorescence was examined with 40x 1.3 NA or 63x 1.2 NA objectives in Leica SP 5X MP confocal laser scanning microscope, CLSM (Leica Microsystems, Heidelberg). Images and overlays were analyzed in Leica software and exported as TIFF files to CorelDraw for composite picture. Except for cropping, no image manipulation was used.

### In vivo animal experiments

The experiments were performed on male Wistar rats and surgical procedures were similar to those described earlier for mice [31]. Briefly, animals were anaesthetised with gas isoflurane and placed on a heated surgical table and maintained at 38^o^ C. The jugular vein was cannulated and thereafter anaesthesia was maintained with intravenous injection of 2 mg/100 g animal pentobarbital hourly or as needed. The abdomen was opened, and the proximal end of the bile duct was ligated. The pancreas and the common pancreatic bile duct was located and cannulated with a polyethylene cannula. The pancreatic juice was collected every 15 minutes. First, the basal secretion was collected for 30 min, and then secretion was stimulated with constant intravenous infusion (0.03 ml/min) of secretin (10 pmol/min/animal). Pancreatic juice samples were collected on ice into weighted vials; secretion rates were calculated and corrected for the animal weight. Pancreatic juice samples were stored at -20^o^ C for further analysis. At the end of the experiment, animals were euthanized with pentobarbital overdose and the samples of stomach contents were collected.

### Administration of PPI

In acute experiments a single dose of omeprazole and SCH28080 were administrated by the intravenous injection through the jugular vein. Omeprazole was dissolved in 40% polyethylene glycol (PEG 400) [32] and SCH28080 was dissolved in 0.4% methylcellulose-saline suspension [33]. The doses were chosen according to the previous studies and injected two hours before collecting pancreatic secretion [33,34]. Omeprazole was given in doses of 5 mg/kg animal and 20 mg/kg animal; SCH28080 was given in 10 mg/kg animal. In matched controls, animals were injected with appropriate vehicle solutions. In the long-term treatment study, animals were treated daily by subcutaneous injections of either 5 mg/kg omeprazole or vehicle (40% PEG) for 30 ± 2 days. The final dose of omeprazole was given a day before the planned operation.

### pH and ion concentrations in pancreatic juice

To avoid contaminations from basal and bile secretion for pH and ion measurements, the first 3 samples were excluded from analysis. The pancreatic juice samples were equilibrated with 5% CO_2_/air for 30 min and the pH was measured using a glass pH combination electrode (Hanna Instruments, nr. HI 1083). HCO_3_^-^ concentrations were calculated using the Henderson-Hasselbalch equation. In order to determine whether PPIs were working as predicted in rats, the pH of stomach contents was also measured, similar to published studies [35]. Stomach contents were centrifuged to obtain the liquid fraction, which was diluted with distilled water at 1:1 ratio and the pH was measured. The following analyses were performed on pancreatic juice samples. Concentrations of Cl^-^ were determined using QuantiChrom Chloride Assay Kit according to the supplier guidelines (Bioassay Systems). Samples were diluted 10x, transferred to 96 microwell plate together with Chloride Assay reagent, absorption at 610 nm was measured and Cl^-^ concentrations were calculated from the standard curve. Na^+^ and K^+^ concentrations were measured using FLM3 flame photometer (Radiometer Copenhagen). Lactate concentrations were measured using Lactate Assay kit (Sigma Aldrich) and phosphate concentrations were measured using QuantiChrom Phosphate Assay Kit (Bioassay Systems), following manufacturer’s instructions.

### Statistics

For real-time PCR, relative quantification (2^-ΔΔCt^) was used, where Ct denotes the threshold cycle. The level of transcripts were normalized to house-keeping genes (ΔCt) and then normalized to the expression in Capan-1 cells (ΔΔCt). The protein level was normalized to β-Actin and then to the Capan-1 protein level. The differences in gene and protein expression were tested using one way analysis of variance (ANOVA) in Sigma Plot 11 and P<0.05 accepted as statistically significant. Data from functional measurements (pH_i_ and pancreatic secretion) are presented as original recordings and summaries showing the mean values ± standard error of mean, SEM. Control and test pH_i_ measurements were made on the same cell, and *n* refers to measurements on different batches of cells. Paired Student´s *t* test was applied. For ion concentration graphs, raw data was bound into 1 μl/min-kg intervals and each bullet shows the mean value ± SEM in secretion rates (x-axis) and ion concentrations (y-axis). Statistical analysis on data obtained from animal experiments was performed using Student′s *t*-test and ANOVA, and *P* < 0.05 was accepted as statistically significant and denoted with *asterisks*.

## Results

### Human duct cells express gastric and non-gastric H^+^/K^+^-ATPases

**Fig 1:**
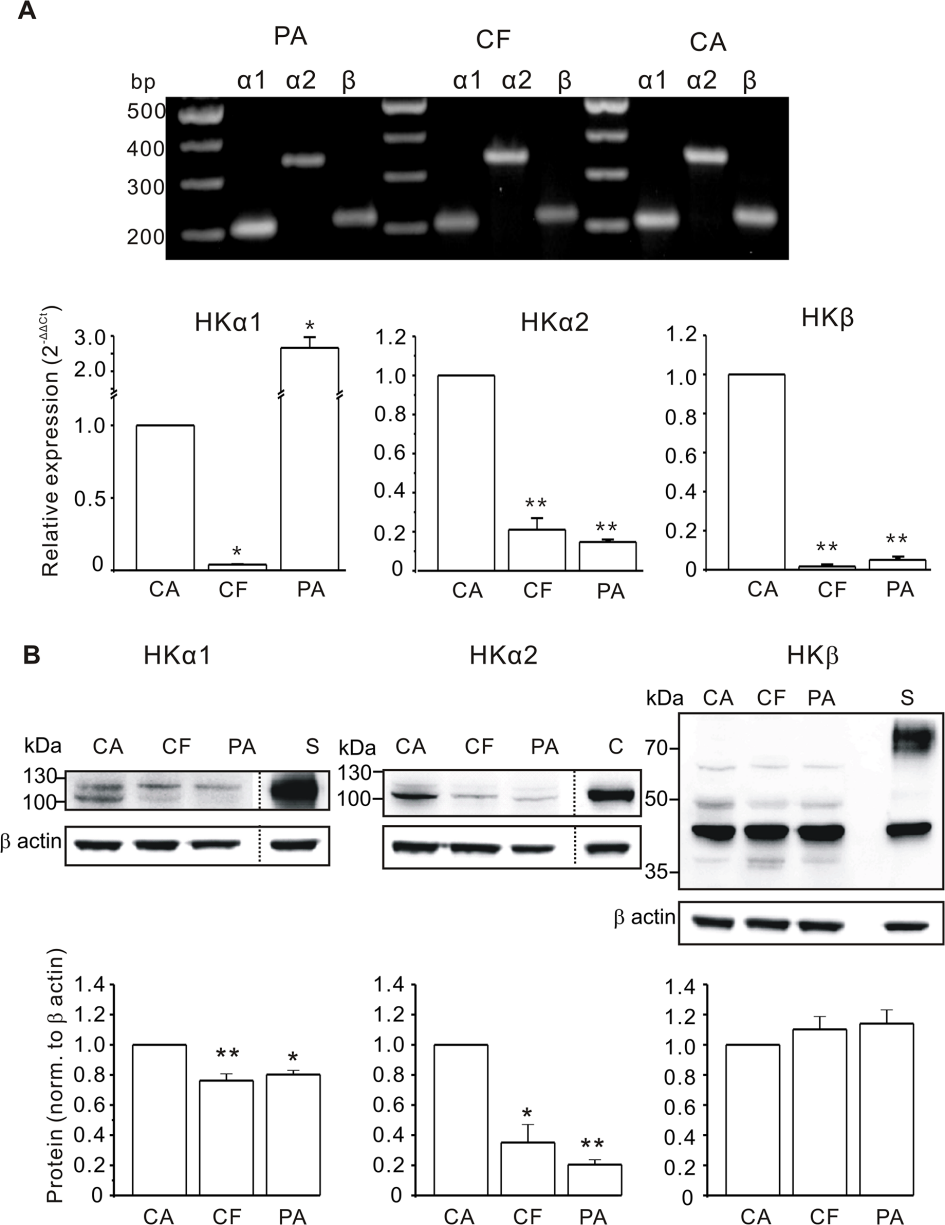
Expression of H^+^/K^+^-ATPases in human pancreatic duct cell lines Capan-1 (CA), CFPAC-1 (CF) and PANC-1 (PA). **A:** RT-PCR analysis of gastric H^**+**^/K^**+**^-ATPase α (HKα1, 200 bp), non-gastric H^**+**^/K^**+**^-ATPase α (HKα2, 339 bp) and β (HKβ, 136 bp) subunits. Representative gels for at least three independent experiments. Real time PCR was used to evaluate the relative expression (2^**-ΔΔCt**^). The house keeping genes 18S ribosomal RNA (18SrRNA), β-actin, β glucuronidase (GUSB) and glutaminyl-tRNA synthetase (QARS) were used and expression in Capan-1 cells was set to be 1. The graph shows data for three experiments (mean±SEM). Significance of expression was tested by one-way ANOVA using the value 2^**-ΔCt**^. **B:** Western blot on cell lysates from duct cell lines, as well as control tissues—mouse stomach (S) and colon (C). Antibodies against gastric H^**+**^/K^**+**^-ATPase α subunits (HKα1, Abcam EPR12251), non-gastric H^**+**^/K^**+**^-ATPase α subunits (HKα2, Sigma, HPA039526) and gastric H^**+**^/K^**+**^-ATPase β subunits (HKβ, Sigma A274) were used. Loading control was β-actin detected at 43 kDa. All lanes were loaded with 60 μg of protein. Stomach and colon gels were run separately. Lower bargraphs show expression of the subunits normalized to actin: the bands at 115 kDa (HKα1); 100 kDa (HKα2) and 45 kDa (HKβ) were used. Data is from 3–4 independent experiments and * indicates *P*<0.05 and ** *P*<0.001 compared to Capan-1.

Human pancreatic duct adenocarcinoma cell lines Capan-1, CFPAC-1 and PANC-1 are commonly used as human pancreatic duct models to study the expression and function of different ion transporters. Capan-1 and PANC-1 cells express functional CFTR, while CFPAC-1 cells have F508 deletion in CFTR and thus the protein expression and function are defect. We used the three cell lines for RT-PCR and results are shown in Fig 1A. We found expression of the gastric H^+^/K^+^-ATPase α subunit (HKα1, 200 bp) and the β subunit (HKβ, 136 bp), as well as the non-gastric H^+^/K^+^-ATPase α subunit (HKα2, 339 bp) in all cell lines. Real time PCR analysis is also shown in Fig 1. Expression levels of transcripts for HKα1, HKβ and HKα2 subunits among different cell lines were compared with respect to Capan-1 cells.

Expression of gastric and non-gastric H^+^/K^+^-ATPases on protein levels was determined in the three cell lines and with protein extracts from the mouse stomach and colon as positive controls (Fig 1B). The HKα1 band at ~115 kDa was detected in cell lysates of the three cell lines and in the stomach. In addition, in Capan-1 cells there was a noticeable band at ~100 kDa. The HKα2 as well as HKβ were also detected in all cell lines. For HKα2, a band at ~100 kDa was detected in colon and in the three duct cell lines. A weaker band at ~115 kDa was also detectable. Similar results were also observed in the rat pancreas [14]. For the HKβ subunit the expected band size for the core protein is 35 kDa, and bands at higher sizes indicate glycosylated subunits [36], most prominently glycosylated in the stomach sample. In pancreatic duct cell lysates, we detected a band with highest intensity at about 40 kDa, similar to the stomach, and also seen in the rat pancreas [14], and this may indicate lower degree of glycosylation of the subunit. Additionally, higher bands from 50 to 65 kDa were also observed in all cell lines.

### Localization of H^+^/K^+^-ATPases in human pancreatic duct cell lines and human pancreas

**Fig 2:**
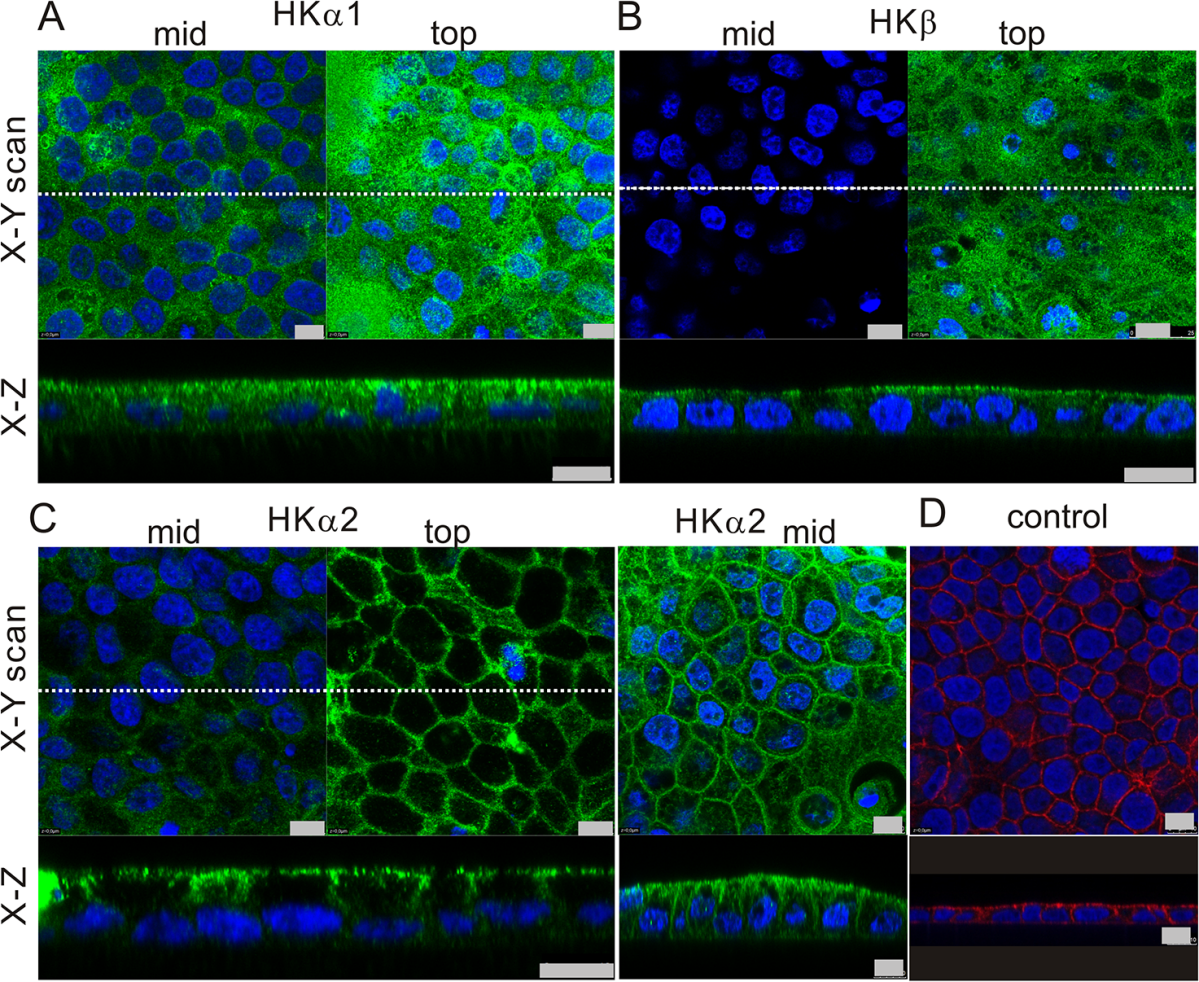
Immunolocalization of H^+^/K^+^-ATPases in Capan-1 cells grown on permeable membranes. **A**: The gastric H^**+**^/K^**+**^-ATPase α subunit (HKα1) was labeled with Calbiochem 119101 (polyclonal, against HKα1C-terminal) and Alexa 488 (green). **B**: The gastric H^**+**^/K^**+**^-ATPase β subunit (HKβ) was detected with Sigma A-274 (2G11, anti HKβ, monoclonal) and Alexa 488 (green). **C**: The non-gastric H^**+**^/K^**+**^-ATPase α subunit (HKα2) was stained with non-gastric HKα2 antibody (C384-M79) and Alexa 488 (green) Three pairs of images are shown. **D**: Example of a control image without primary antibodies but with F-actin marker (phalloidin Texas red). DAPI was used to stain the nucleus (blue). All bars are 10 μm, and images from at least 10 independent experiments are presented as both x-y and x-z scans. In x-y scan the left images are taken mid-way through the monolayer, the right images are taken close to the apical membrane. Dotted lines in x-y scans indicate where the x-z scan was taken.

**Fig 3:**
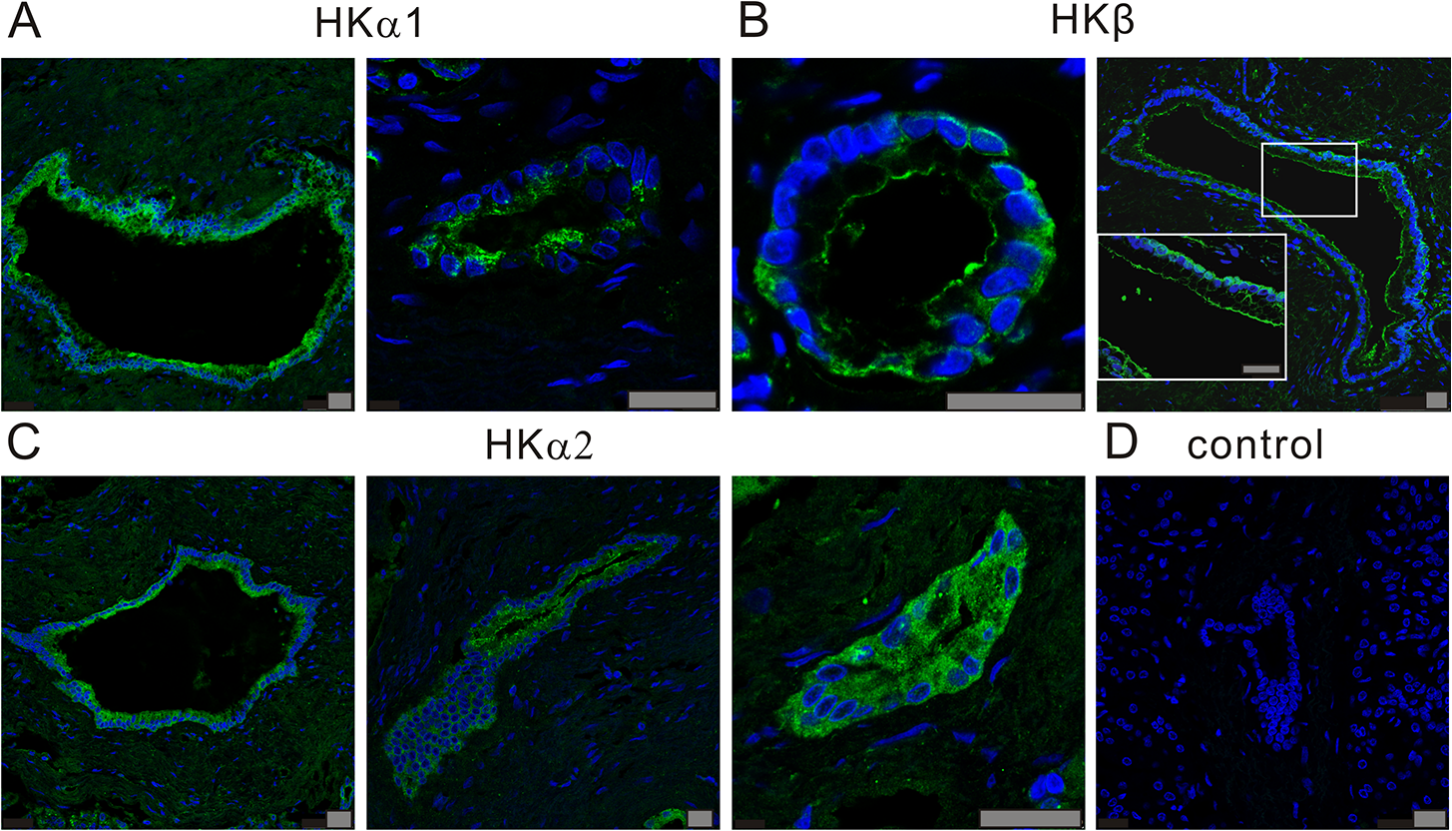
Immunolocalization of H^+^/K^+^-ATPases in sections of human pancreas. **A**: The gastric H^**+**^/K^**+**^-ATPase α subunit (HKα1) was labeled with Calbiochem 119101 (polyclonal, against HKα1C-terminal) (image 1 and 2) and Calbiochem 119102 (polyclonal, against HKα1 N-terminal) and Alexa 488 (green); **B**: The gastric H^**+**^/K^**+**^-ATPase β subunit (HKβ) was stained with Sigma A-274 (2G11, anti HKβ, monoclonal) and Alexa 488 (green). **C:** The non-gastric H^**+**^/K^**+**^-ATPase α subunit (HKα2) was stained with non-gastric HKα2 antibody (C384-M79) and Alexa 488 (green). **D**: Example of a control image without primary antibodies. DAPI was used to stain the nucleus (blue). All bars are 25 μm, and images are from 3 independent experiments and show localization in ducts of various sizes.

The expression and localization of H^+^/K^+^-ATPases were further analyzed using immunofluorescence and confocal microscopy. Immunoreactivity for HKα1, HKα2 and HKβ subunits was observed in the three duct cell lines (data not shown), but here we focus on Capan-1 cells, which form pancreatic duct epithelia with characteristic ion transporters when grown on permeable membranes [4]. Fig 2 shows images of H^+^/K^+^-ATPase α1, β or α2 subunits on non-stimulated cells. For all subunits some immunoreactivity was detected intracellularly, e.g. in vesicles, but we also observed expression of the pumps on the plasma membranes. The gastric HKα1 subunit was most strongly expressed on and close to the luminal membranes (Fig 2A). The gastric HKβ subunit was predominantly found on the luminal side of the epithelium (Fig 2B). The non-gastric HKα2 subunit localized to the luminal, and importantly also to the lateral membranes of the epithelium (Fig 2C). Whole human pancreas sections showed similar staining to Capan-1 cells. Fig 3 shows images of ducts of various sizes, and surrounding pancreatic acini, which did not stain with HK antibodies. The gastric HKα1 localized on or close to the luminal membrane and sub-membrane vesicles in human pancreatic ducts. The gastric HKβ subunit was clearly localized to the luminal membrane, and more diffusely, possibly in vesicles, proximal to the basal plasma membrane of pancreatic duct cells. The non-gastric HKα2 subunit was detected intracellularly, as well as on the plasma membranes.

### Intracellular pH in Capan-1 cells is sensitive to proton pump inhibitors

**Fig 4:**
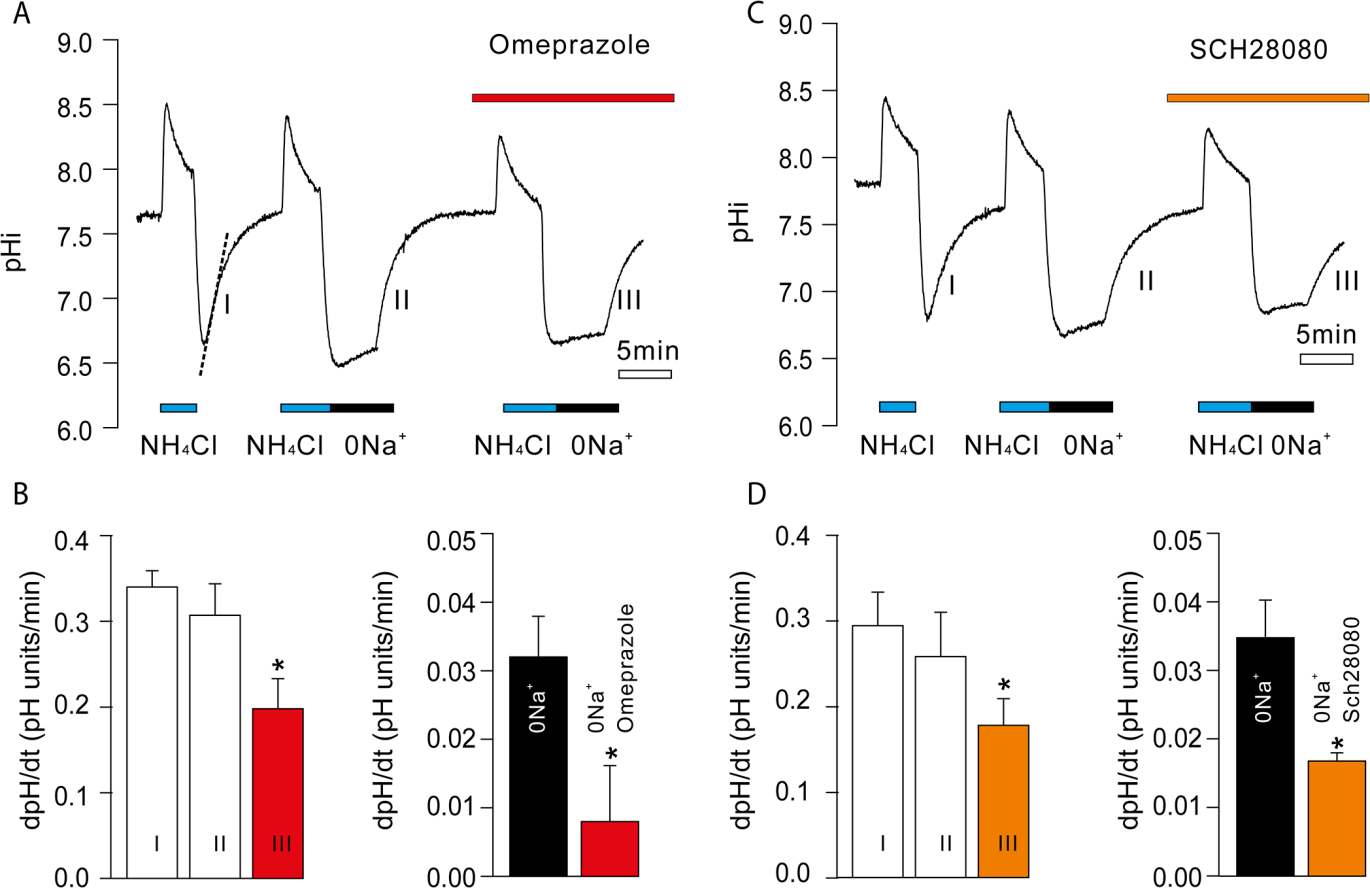
Effect of H^+^/K^+^-ATPase inhibitors on pH_i_ recovery. **A:** Representative recording of a pH_**i**_ measurement in Capan-1 cells challenged with ammonium pulse in Na^**+**^-containing physiological buffer, then in a Na^**+**^-free buffer with or without Na^**+**^ with omeprazole (10 μM). Dotted lines show the slope of the pH_**i**_ recovery from acidosis, i.e. dpH/dt. The pHi recovery was determined when cells were returned to control conditions (periods I, II and III) and in periods with Na^**+**^-free buffer +/- proton inhibitor. **B:** Summary of recovery rates expressed as dpH/dt upon return to Na^**+**^-containing buffer (first three bars) and in Na^**+**^-free buffer (next two bars) (n = 6). **C, D**: similar representative recording and summary for SCH28080 (10 μM, n = 5). Cells were stimulated with secretin (10^**–9**^ M) and buffers were HCO_**3**_^**—**^free in order to eliminate contribution of HCO_**3**_^**-**^ transporters. Bars show paired measurements as means ± SEM. Asterisks indicates P < 0.05.

In order to evaluate the function of H^+^/K^+^-ATPases, we applied a method common to study HCO_3_^-^/H^+^ transport, i.e. monitoring pH_i_ recovery from an acid load, i.e. the NH_4_^+^/NH_3_ pre-pulse technique (Fig 4A and 4C). In order to eliminate the contribution of Na^+^ and/or HCO_3_^-^ transporters to pH_i_ recovery, we used Na^+^- and/or HCO_3_^—^free solutions for bath perfusion. Capan-1 cells were continuously stimulated with secretin (10^–9^ M) during the experiments, to imitate stimulated ductal epithelium [37]. The pH_i_ recovery rate of secretin-stimulated Capan-1 cells in HCO_3_^—^free physiological buffers was 0.313±0.018 pH units/min, and it was significantly reduced to 0.036±0.003 pH units/min in the absence of extracellular Na^+^ (n = 15). However, Capan-1 cells were still able to defend pH_i_ changes even without HCO_3_^-^ transporters and Na^+^/H^+^ exchangers. Importantly, this Na^+^ independent pH_i_ recovery was reduced by H^+^/K^+^-ATPases inhibitors (Fig 4B and 4D). The gastric H^+^/K^+^ pump inhibitor omeprazole inhibited 75% of the Na^+^ independent pH_i_ recovery (n = 6), while SCH28080 reduced the Na^+^ independent pH_i_ recovery by 52% (n = 5). In addition, PPIs also reduced pH_i_ recovery when cells were returned to control Na^+^ containing buffer (Fig 4 phase III vs. I).

### Proton pump inhibitors reduce pancreatic secretory rates

**Fig 5:**
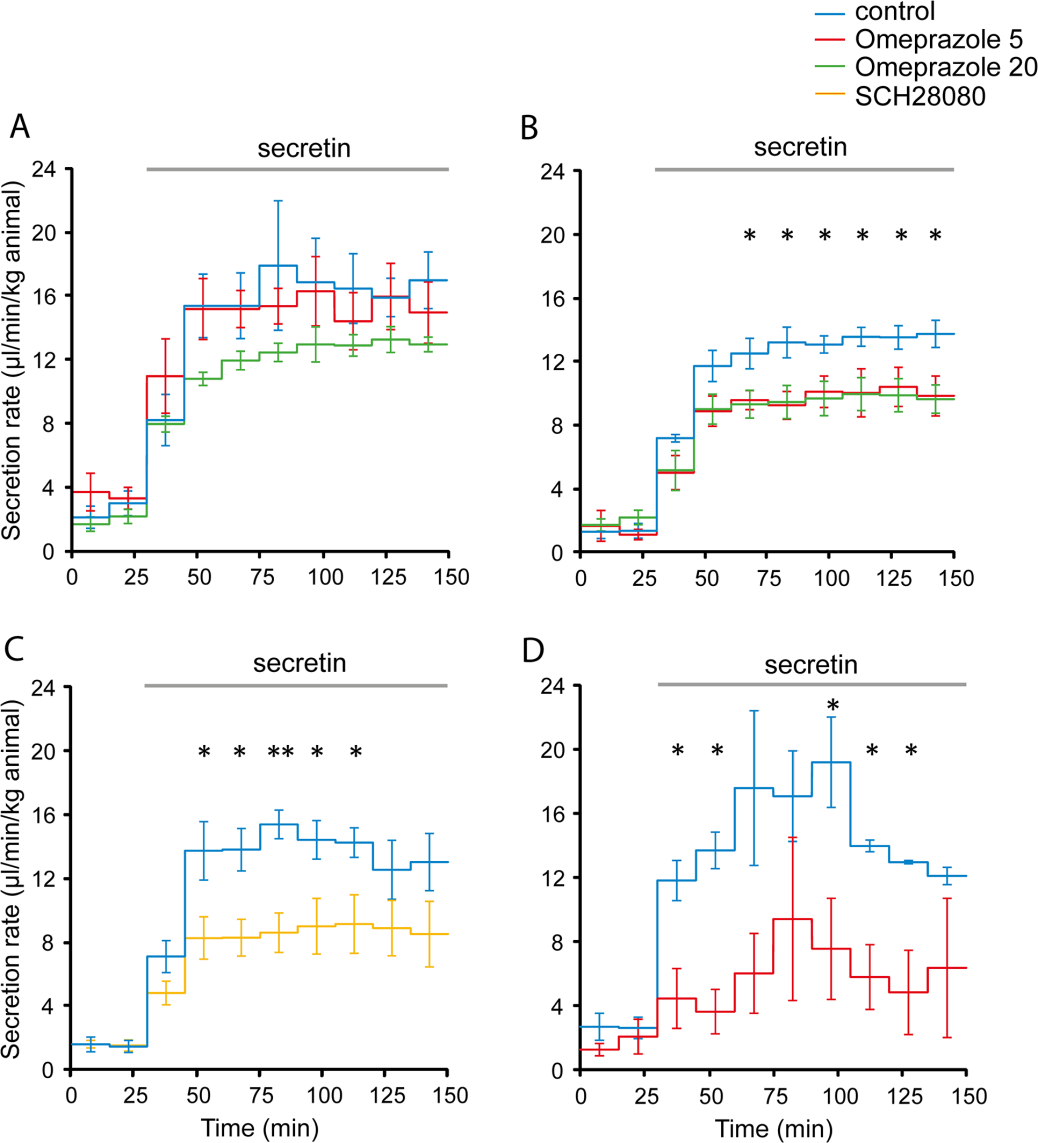
Effects of proton pump inhibitors on pancreatic secretion rates in rats. Controls are represented as blue line and in each graph n is 6, 4, 7 and 4 respectively. **A**: Effects of 5 mg/kg (red line, n = 4) and 20 mg/kg omeprazole (green line, n = 6) on non-fasted rats. **B**: Effects of 5 mg/kg (red line, n = 4) and 20 mg/kg omeprazole (green line, n = 4) on fasted rats. **C**: Effect of 10 mg/kg SCH28080 (yellow line, n = 6) on fasted rats. **D**: Effects of long term (30 days) daily administration of 5 mg/kg omeprazole (red line, n = 4). Secretory rates are represented as mean values ± SEM. * indicates *P*<0.05, ** P<0.01.

The crucial question to answer now was whether the H^+^/K^+^-pumps contribute to pancreatic secretion. Therefore, the following acute and long-term *in vivo* experiments with proton pump inhibitors were performed on rats. In the first series of experiments, animals had free access to food prior to surgery. Omeprazole was given intravenously two hours before pancreatic secretion was induced with secretin. Two doses of omeprazole (5 mg/kg and 20 mg/kg animal) were tested and the results are shown in Fig 5A. Clearly, 5 mg/kg omeprazole had no significant effect on pancreatic secretion compared to the vehicle infusion (n = 4 and 6, respectively). High dose of omeprazole (20 mg/kg animal) had a tendency to reduce pancreatic secretion rate by about 30% (n = 6), however, statistical significance was not reached with these number of experiments. Pancreatic juice contained relatively high content of secreted proteins, i.e. 29 g/l, which would indicate enzyme secretion from acini. It is well recognized in pancreatic physiology that non-fasted animals have higher fluid and enzyme secretions, due to the endogenous hormones/transmitters that can activate acinar and duct secretions. Therefore, in all following experiments animals were fasted overnight. Fig 5B shows the effect of low and high doses of omeprazole, which now caused significant effects on secretion apparent 30 minutes after stimulation and maintained throughout the experiment. Integrated secretion in the first and second hour after secretin stimulation were 663±42 and 804±39 µl/h/kg in control animals (n = 4), and significantly lower in test animals in the same sample periods, i.e. 484±51 (*P* = 0.018) and 563±68 µl/h/kg (*P* = 0.014) after low dose omeprazole treatment (n = 4), and 487±58 (*P* = 0.027) and 581±60 µl/h/kg (*P* = 0.013) after high dose omeprazole treatment (n = 4). Interestingly, both 5 and 20 mg/kg omeprazole had similar effects, indicating that the maximal effective dose was reached at 5 mg/kg. This dose of omeprazole also effectively inhibited basal gastric acid secretion in rats, though 20 mg/kg dose was required for inhibition of pentagastrin stimulated gastric secretion [34]. In our experiments, we also checked that omeprazole was working on gastric acid secretion by measuring stomach pH. In control animals pH_stomach_ was 4.16±0.08 and in omeprazole-treated animals it was 5.05±0.13 (n = 15, *P* = 1.00×10^–5^).

In order to check the possible contribution of non-gastric H^+^/K^+^ pump to pancreatic secretion, the acute effects of SCH28080 were tested. SCH28080 inhibits gastric pump and it has been reported that in high doses it can also inhibit non-gastric H^+^/K^+^ pumps [20,27,28]. In Fig 5C it can be seen that administration of 10 mg/kg SCH28080 resulted in a significant reduction of secretory rates. During the first hour of secretin stimulation, the secretion was reduced from 739±50 to 438±65 µl/h/kg (*P* = 0.002) and during the second hour from 1174±92 to 501±117 µl/h/kg (*P* = 0.0009), comparing control and treatment groups (n = 7; 6). There seems to be more pronounced inhibition of secretion with SCH28080 than with omeprazole compared to their respective controls. Interestingly, given the dose of inhibitors used in our studies, we would have expected weaker inhibition by SCH28080, due to higher expected ED_50_ values. That is, ED_50_ of 0.8 mg/kg for omeprazole and 3 mg/kg of SCH28080 was determined for rodent gastric function [33,38].

Above experiments show that acute treatment with both types of blockers (for simplicity denoted here PPIs) reduced pancreatic secretion. In order to imitate PPI treatment in humans, it was relevant to investigate the outcome of long-term omeprazole treatment. Animals were treated with omeprazole (5 mg/kg) or vehicle for 30 days and subsequently pancreatic secretion was monitored in anaesthetized animals. The results of the long-term study are represented in Fig 5D. Comparing control and treatment groups, during the first hour of secretin stimulation the secretion was reduced from 906±130 to 356±159 µl/h/kg (*P* = 0.019) and during the second hour from 876±49 to 403±179 µl/h/kg (*P* = 0.036) (n = 4; 4). Together, these data on short- and long-term treatment with PPIs show that they have significant inhibitory effect on pancreatic secretion. Do they also have effects on electrolyte composition of pancreatic juice and can that reveal how the secretion is formed?

### Effect of proton pump inhibitors on pancreatic juice electrolytes

**Fig 6:**
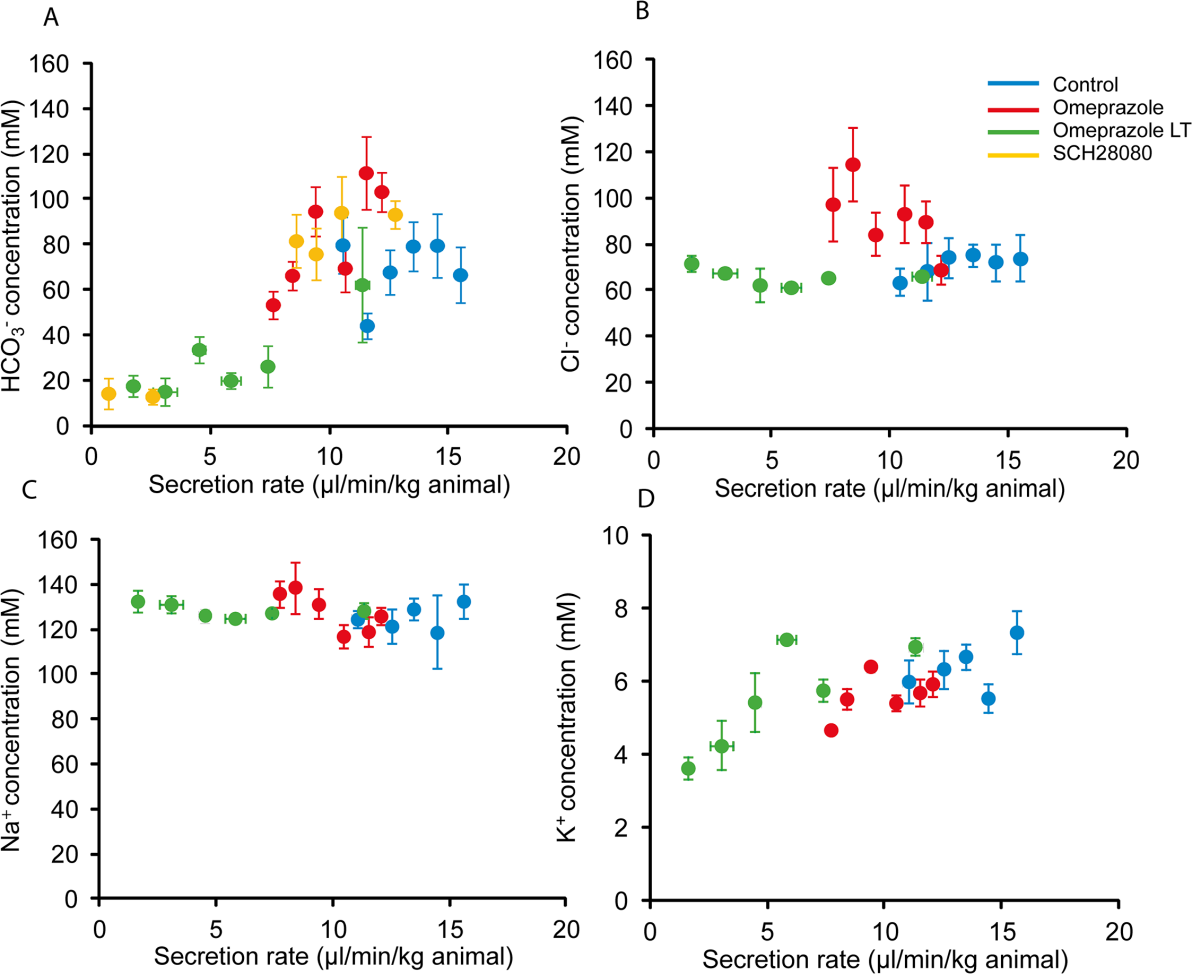
Composition of pancreatic secretions. Relation between pancreatic secretory rates and concentrations of HCO_**3**_^**-**^ (**A**), Cl^**-**^ (**B**), Na^**+**^ (**C**) and K^**+**^ (**D**). Data for control experiments are represented blue, acute omeprazole treatment experiments in red and long-term omeprazole treatment experiments in green. Data for effect of SCH28080 on HCO_**3**_^**-**^ concentrations are also included and marked in yellow symbols. Data are represented as mean value±SEM for 3–15 samples that were binned in 1 µl/min-kg secretion rate intervals.

Results of sample analyses for acute and long-term omeprazole experiments compared to control experiments are given in Fig 6. Fig 6A shows that HCO_3_^-^ concentrations depend on secretory rates. Without inhibitors, in the secretory rate range 10 to 15 µl/min-kg body weight, HCO_3_^-^ concentrations were between 60 to 80 mM. With acute omeprazole treatment, secretion rates decreased and in the secretory range 7.5 to 12.5 µl/min-kg, HCO_3_^-^ concentrations spread between 45 to 90 mM. Prolonged omeprazole treatment resulted in low secretory rates, below 7 µl/min-kg, and HCO_3_^-^ concentrations were around 10 to 20 mM. Also with SCH28080, HCO_3_^-^ concentrations decreased in low secretory rates. Nevertheless, it appears that all data fall into a HCO_3_^-^ excretion curve that follows a characteristic pattern, which is valid for several animal species and humans [14].

Fig 6B shows that Cl^-^ excretion pattern in control and acute omeprazole samples is inversed to that for the HCO_3_^-^ pattern, as expected [1]. However, in samples from the long-term omeprazole treated animals, Cl^-^ concentrations were unexpectedly low and independent of secretory rates. There seemed to be an anion deficit in pancreatic juice, which could have been due to one or more organic or inorganic anions. We determined lactate in the juice and found that concentrations were low in both types of experiments: in control samples they were 0.79 ± 0.07 mM (20 samples form 13 animals) and between 0.60 ± 0.04 mM in PPI samples (40 samples from 18 animals). In addition, we estimated inorganic phosphate concentrations, which were between 1.5 and 3.5 mM in both control and PPI samples and given pH values in secreted pancreatic juice, we estimate that they contribute 2.5 to 6 mequivalents/l.

Fig 6C shows typical Na^+^ excretory pattern that is independent of secretory rates. Most importantly, Fig 6D shows that K^+^ excretion has a positive linear correlation with the secretory rates (r = 0.642 and *P*<0.0001). Notably, at higher secretion rates pancreatic juice K^+^ concentrations are higher than the plasma values.

## Discussion

This is the first study that shows the expression of H^+^/K^+^-ATPase α and β subunits in human pancreatic duct cells on both molecular and functional levels. Furthermore, substantial effect of proton pump inhibitors on the whole pancreas level confirms that H^+^/K^+^-ATPases contribute to pancreatic secretion, though finding of luminally placed ATPases is surprising. These findings may mark a shift in paradigm in our understanding of acid/base transport in the pancreas.

### Various pancreatic duct cells express H^+^/K^+^-ATPases subunits

A number of experimental approaches in the present study show that several human duct cell lines, as well as human pancreatic sections and the whole rat pancreas express gastric and non-gastric H^+^/K^+^-ATPases. First, we observed the expression of both gastric and non-gastric H^+^/K^+^-ATPases on mRNA and protein levels in three different types of human duct cell lines, and these findings agree with those made on the rat pancreas [14]. The differential expression of pumps in the three cells lines may reflect the fact that they are cancer cells with different properties. Nevertheless, Capan-1 cells, which can be grown as monolayers are good models for transepithelial ion transport in human ducts [4,37], and we used these for further functional and immunolocalization studies.

The non-gastric pumps have relatively wide distribution and function in H^+^ and cation homeostasis (see introduction). The targeting of the non-gastric HKα2 subunit to plasma membranes (apical or basolateral) depends on coding motifs and interactions within the subunit, and possibly association with gastric HKβ or β-subunit of the Na^+^/K^+^-ATPase, probably explains localizations to the apical and/or basolateral membranes of epithelia [24,39–42]. The gastric pump has been reported for H^+^ secreting epithelia (see introduction) and its presence in the HCO_3_^-^ secreting epithelium, the pancreas, seemed somewhat peculiar. From a number of expression studies it is known that specific motifs in the HKα subunit and the glycosylated HKβ subunit are required for targeting and functional assembly [36,43,44]. Notably, in Western blot analysis of human material, bands of about 40~65 kDa were detected for gastric HKβ subunit (Fig 1), which might indicate low glycosylation of the protein, also detected in rat pancreatic ducts [14]. Nevertheless, we still observe similar localization of the gastric HKβ subunit with α subunits close to the plasma membrane (Figs 2 and 3). Thus, from molecular considerations one might expect both the gastric and non-gastric H^+^/K^+^-ATPase to be functional. The next question is how their cellular localization explains pancreatic secretion.

### Localization of the H^+^/K^+^-ATPases—possible functions on the duct epithelium

The immunohistochemical data (Figs 2 and 3) shows that the two types of pumps have somewhat different localizations on the pancreatic epithelium. The results for human duct cell lines in particular show that the H^+^/K^+^-pumps (predominantly non-gastric type) are expressed intracellularly and on the lateral membrane of pancreatic ducts. The gastric HKα1 subunits are detected on the luminal membranes and in adjacent intracellular vesicles, resembling those in parietal cells. Also gastric HKβ subunits show most distinct placement on or close to the luminal membranes, indicating that functional gastric H^+^/K^+^ ATPase would be formed. This differential pump distribution is similar to that observed in the rat pancreatic ducts [14]. Although immunolocalization shows some overlap between gastric and non-gastric pump distribution, the most interesting question is whether the basolateral and luminal pumps would have different functions.

Let us address the basolateral pumps. The HCO_3_^-^ driven fluid secretion can be achieved by basolateral HCO_3_^-^ transporter loading duct cells with HCO_3_^-^ or transport of H^+^ across the basolateral membrane and intracellular HCO_3_^-^ generation. Thus, the H^+^/K^+^-ATPases localized on the lateral and basal membranes would fulfill HCO_3_^-^ secretion by pumping H^+^ to the basolateral side, leaving HCO_3_^-^ for luminal transport. Both the pH_i_ data and pancreatic secretion data are consistent with above considerations (Figs 4 and 5).

The second observation that H^+^/K^+^-pumps (notably the gastric type) are also on the luminal membrane of pancreatic ducts seems at first perhaps unusual. However, similar pumps (vacuolar type proton pumps) are found in fish intestine and insect midgut that also secrete HCO_3_^—^rich fluid [45,46]. Also airway epithelia express proton pumps (and show sensitivity to vacuolar, gastric and non-gastric pump inhibitors) on the luminal membrane [47–51], though compared to pancreas, their net HCO_3_^-^ secretion is lower and pH of airway surface liquid layer is < pH 7.4 [52]. The function of luminal proton pumps is unclear.

Let us consider possible functions for the luminal proton pumps. We propose that the luminal H^+^/K^+^-ATPases participate in a protection mechanism in the base secreting epithelium. One may draw inspiration from the mucus-bicarbonate barrier found in stomach and duodenum [1], where the barrier provides a near-neutral pH and protection at the epithelial surfaces [53–55]. In pancreas we have the reverse situation. Human pancreatic duct cells are able to secrete up to 140 mM HCO_3_^-^ and luminal pH values are above 8 [1,56,57]. Secretion of H^+^ may provide protection of luminal epithelial surface. Additionally, several mucin genes have been identified in pancreatic duct cells and pancreatic mucins would be relevant in epithelial protection, and altered expression pattern of mucins is one of the important factors in development and drug resistance in pancreatic cancer [58–60]. In physiological context, we propose that H^+^ secretion and mucus could provide protection against luminal base, a so called “pancreatic mucus acid barrier” [1]. In addition, H^+^/K^+^-ATPases would serve to recirculate secreted K^+^ (see below).

### Pancreatic secretion—H^+^/K^+^-ATPases on integrative level and effect of proton pump inhibitors

The observation that PPIs and P-CABs inhibit secretin-evoked pancreatic secretion in *in vivo* rat studies shows that H^+^/K^+^-pumps are in general involved in pancreatic duct secretion. Rat pancreatic secretion is sensitive to omeprazole, as even the low doses were effective (Fig 5). Omeprazole, the acid-activated pro-drug, would inhibit the gastric pump, while SCH28080, which competitively binds to the K^+^ site, could, most likely, inhibit both gastric and non-gastric pumps and seems more effective given the dose used in our experiments and published ED_50_ values [33,38]. However, clear functional and pharmacological distinction between the two types of pumps was not possible in our study. Nevertheless, the most important observation is that PPIs had significant effects. In pH_i_ studies, we acidified the pro-drug and thus the activated form of omeprazole would be formed. However, in animals, omeprazole would have to be activated by the acid environment, and the simplest theory is that this would be by the pancreatic H^+^/K^+^-ATPases directly. This might be somewhat analogous to what happens in the stomach, and thus then provides further evidence for the pumps.

The long-term experiments on rats were performed to find whether omeprazole had cumulative effect on inhibition of pancreatic secretion, or whether animals adjusted to the treatment and regained normal secretory rates. Clearly, the first was the case and pancreatic secretion was further reduced by long-term PPI treatment.

In addition to secretory rates, the excretory electrolyte curves indicate underlying secretory mechanisms. First of all, HCO_3_^-^ excretory curve follows predicted relation with secretory rates. The fact that PPIs do not significantly alter the curve/form indicates that H^+^/HCO_3_^-^ transport drives the fluid transport and thus secretion. Interesting point to recall here is that rodents, other animals and humans do show similar HCO_3_^-^ excretory curves, indicating similar underlying mechanisms [1,14]. The Cl^-^ excretory curves are inversed to those of HCO_3_^-^, which may be due to Cl^-^/HCO_3_^-^ exchange, or other more complex mechanisms [1], and we cannot explain anion deficit at low secretory rates.

Regarding the cation excretion, Na^+^ is not dependent on secretory rates. Similar was thought to be the case for K^+^ excretion. However, our data shows that there is a positive relation between K^+^ and secretory rate, and moreover that K^+^ concentrations are higher than plasma values (Fig 6). Similar higher K^+^ concentrations were also reported in a few early studies [61–63]. Increased K^+^ concentrations in pancreatic juice are most likely due to K^+^ secretion into duct lumen via luminal K^+^ channels, such as K_Ca_3.1, K_Ca_1.1 or KCNQ1 [7,64]. These channels are expressed on luminal membranes and their activation would provide increased driving force for secretion as already predicted earlier [1,6,7]. In that context, it is reasonable to envisage that the luminal H^+^/K^+^-ATPase could thereby operate and in fact serve to recirculate or salvage K^+^ from the lumen, and would explain the observation that H^+^ and K^+^ transport across the luminal membrane is inversely related.

### Clinical implications

Proton pump inhibitors (PPIs) are used widely in clinical practice as one of the treatments for various acid-peptic disorders [65]. As adjuvant therapy PPIs are also prescribed to patients with pancreatic diseases, such as cystic fibrosis and pancreatitis [66]. Moreover, they are also suggested as an adjuvant therapy for patients with type 2 diabetes, supposedly because they elevate plasma gastrin and thereby improve insulin secretion [67,68]. Our acute and long-term experiments on rats show that pancreatic secretion is significantly inhibited by PPIs. Therefore, considering the parallels between H^+^/K^+^-ATPases expression in rodent and human pancreatic epithelia, it would be important to re-consider effect of PPIs on human pancreatic function. Furthermore, our recent data mining analyses indicate that on the mRNA level *ATP4A* and *ATP4B* are down-regulated in pancreatic cancer [69]. Therefore, the role of H^+^/K^+^-ATPases, alongside with other acid-base transporters, should be considered in potential development of deregulated acid-base homeostasis in pancreatic ductal adenocarcinoma.

### Conclusions

In conclusion, we find that pancreatic ducts express both gastric and non-gastric H^+^/K^+^-ATPases. These pumps are functional in human duct cells and in rat pancreas on the whole organ level where they contribute to secretion as demonstrated by PPIs. The laterally and basolaterally expressed H^+^/K^+^-ATPases would export H^+^ out of the cell, leaving HCO_3_^-^ for luminal transport and duct secretion. Localization of the H^+^/K^+^-ATPases to the luminal membrane is a change in paradigm, and we propose that they may contribute to the protective buffer zone and K^+^ recirculation. Lastly, the solid evidence that we provide for the H^+^/K^+^-ATPases in pancreas calls for re-evaluation of the use of PPIs as they will not only affect stomach secretion but also pancreatic secretion—contrary to what may be wished.

## Supporting Information

S1 Fig Original blots. (PDF)
