# Supplementary material for: Proton Pump Inhibitors Inhibit Pancreatic Secretion: Role of Gastric and Non-Gastric H+/K+-ATPases
Source: PLoS One. 2015 May 18;10(5):e0126432. doi: 10.1371/journal.pone.0126432 (PMC4436373; doi:10.1371/journal.pone.0126432)

Fig. 1A

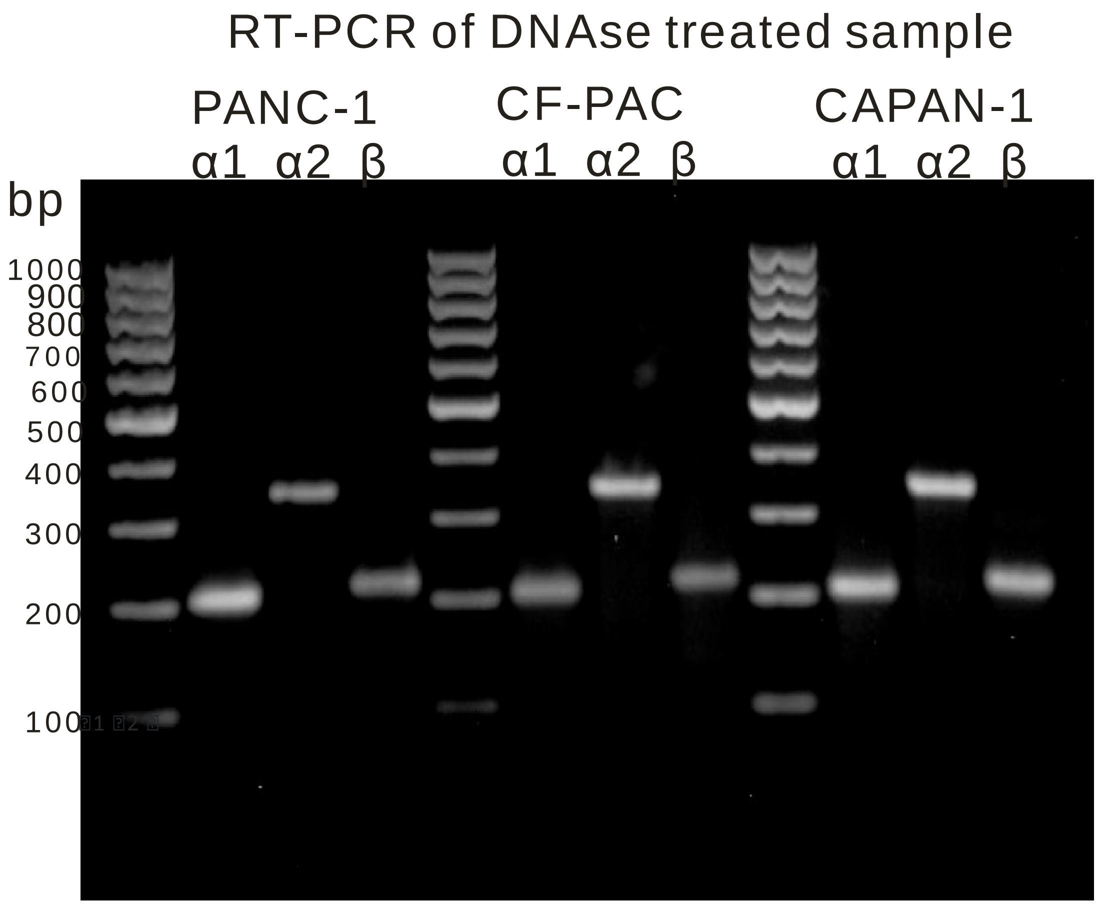

α1=alpha subunit of gastric HK pump (200bp)
α2=alpha subunit of non-gastric HK pump(339bp)
β=beta subunit of gastric HK pump(213bp)

RNA template was treated with DNAse
to get rid of DNA contamination

Fig. 1 B 3 parts

HKα1

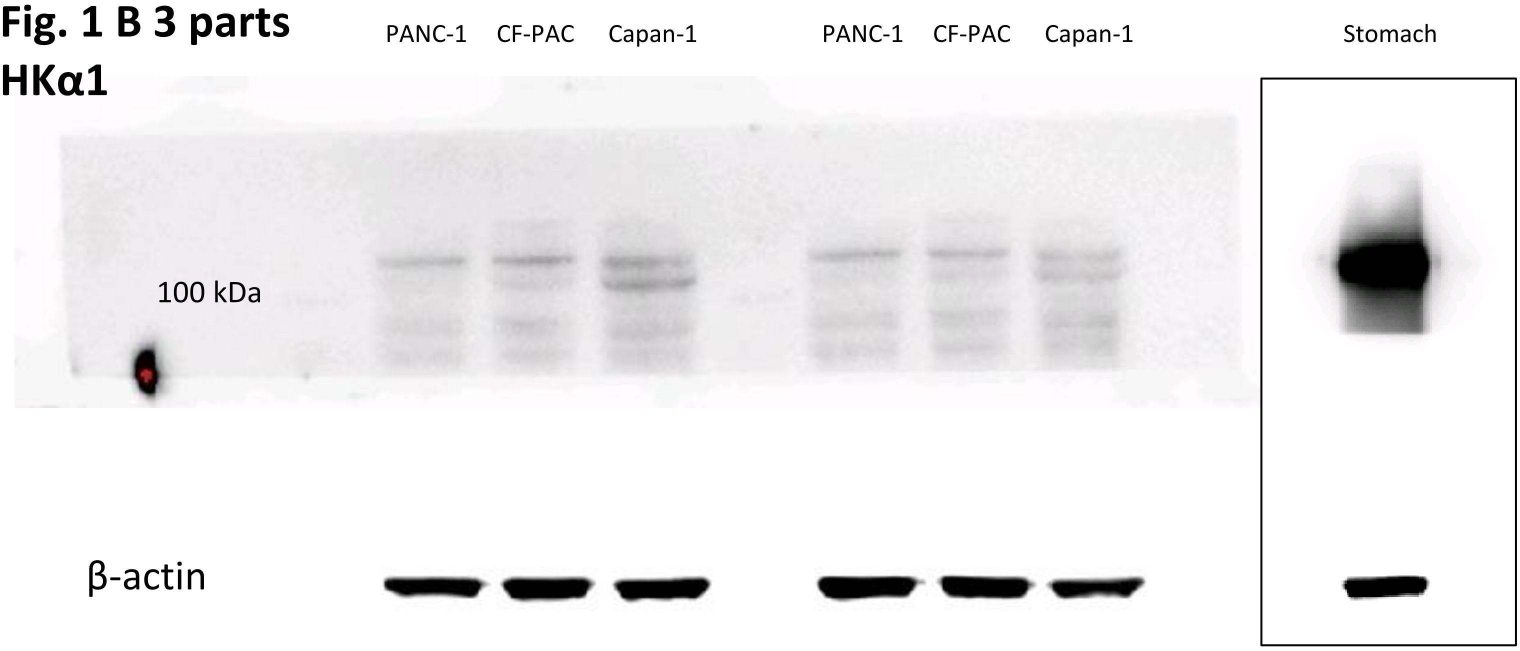

Proteins run in duplicate with actin // Stomach and colon run separately due to the different exposure time

HKα2

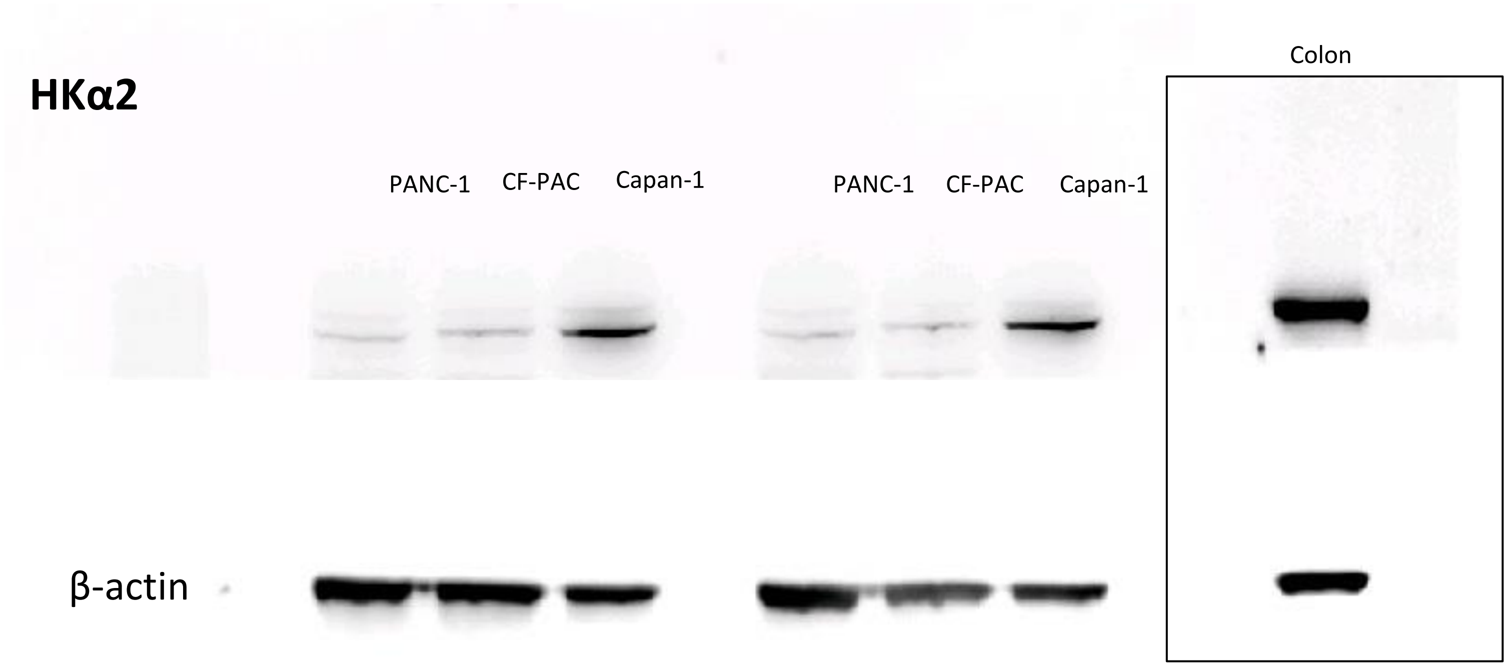

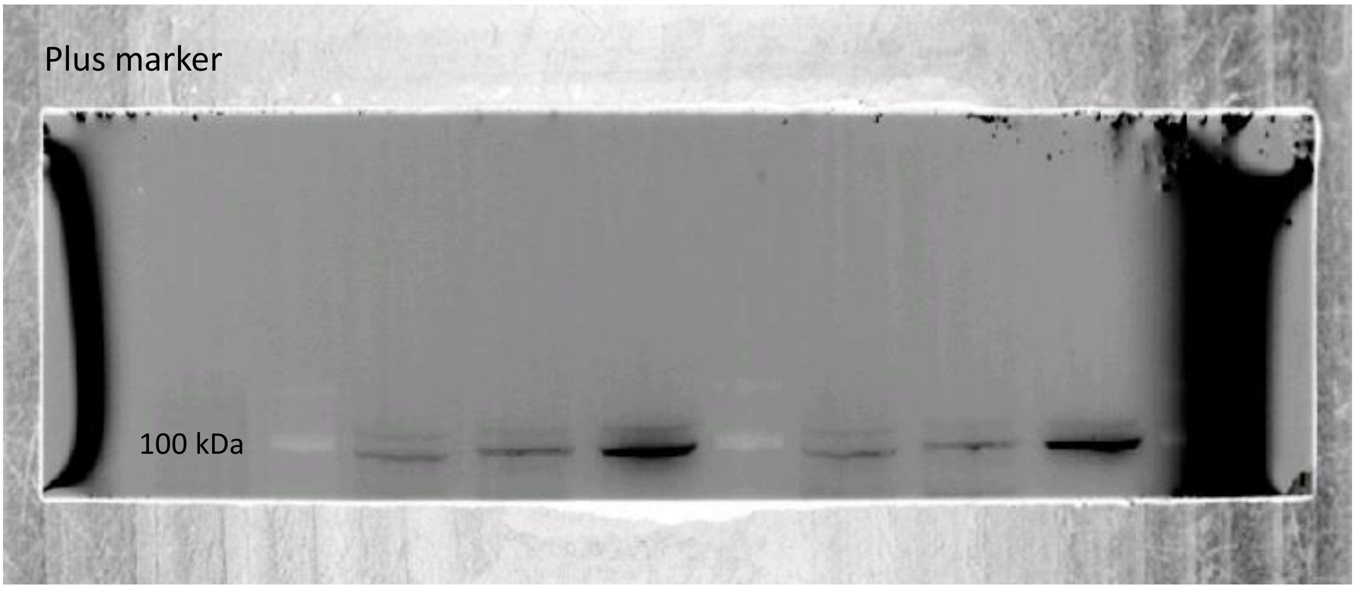

HK β

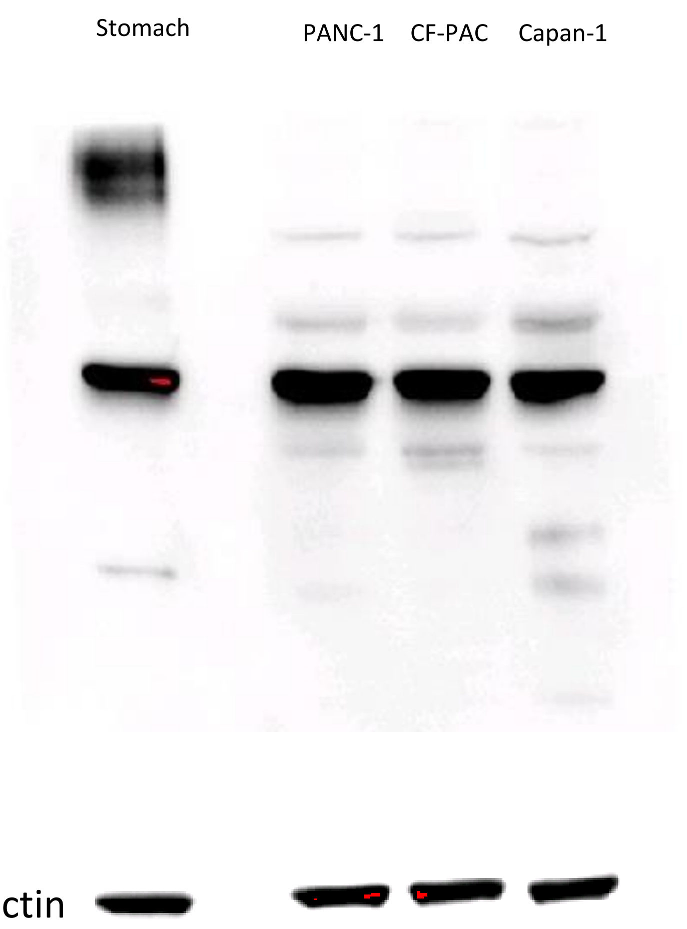

Plus marker

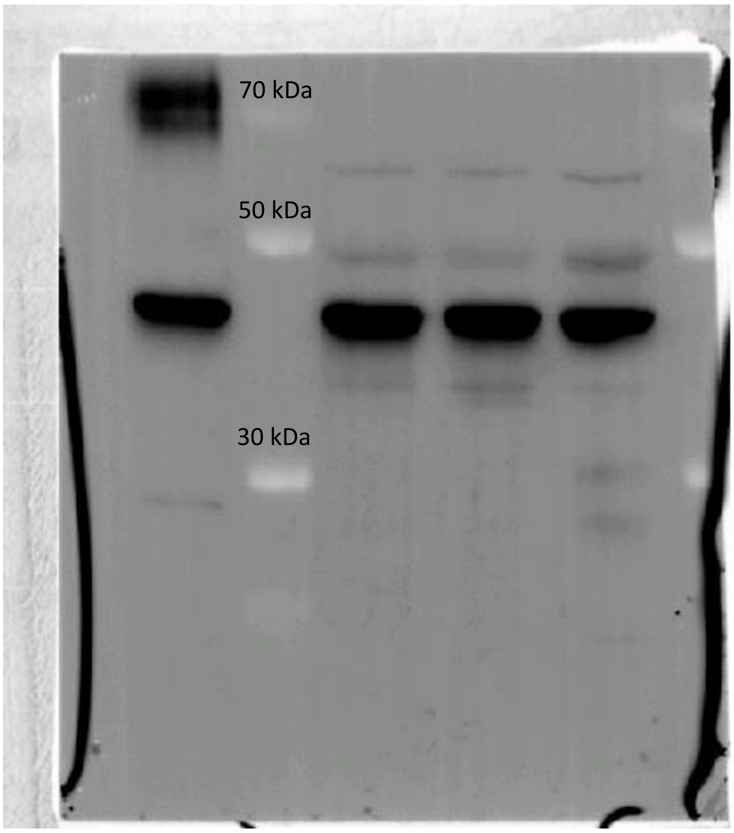

Supplement: S1 Fig — (PDF) [file pone.0126432.s001.pdf]
